# Retargeting of Viruses to Generate Oncolytic Agents

**DOI:** 10.1155/2012/798526

**Published:** 2011-11-14

**Authors:** M. H. Verheije, P. J. M. Rottier

**Affiliations:** ^1^Pathology Division, Department of Pathobiology, Faculty of Veterinary Medicine, Utrecht University, Yalelaan 1, 3584 CL Utrecht, The Netherlands; ^2^Virology Division, Department of Infectious Diseases & Immunology, Faculty of Veterinary Medicine, Utrecht University, Yalelaan 1, 3584 CL Utrecht, The Netherlands

## Abstract

Oncolytic virus therapy is based on the ability of viruses to effectively infect and kill tumor cells without destroying the normal tissues. While some viruses seem to have a natural preference for tumor cells, most viruses require the modification of their tropism to specifically enter and replicate in such cells. This review aims to describe the transductional targeting strategies currently employed to specifically redirect viruses towards surface receptors on tumor cells. Three major strategies can be distinguished; they involve (i) the incorporation of new targeting specificity into a viral surface protein, (ii) the incorporation of a scaffold into a viral surface protein to allow the attachment of targeting moieties, and (iii) the use of bispecific adapters to mediate targeting of a virus to a specified moiety on a tumor cell. Of each strategy key features, advantages and limitations are discussed and examples are given. Because of their potential to cause sustained, multiround infection—a desirable characteristic for eradicating tumors—particular attention is given to viruses engineered to become self-targeted by the genomic expression of a bispecific adapter protein.

## 1. Introduction 

Cancer is one of the major health problems of our times. Though the prognosis for people diagnosed with, at least some forms of, cancer has increased considerably, it is more typical a disease of which treatment is initially effective, to be followed later by an irreversible and eventually fatal relapse. Already for decades, cancer treatment is based on three types of approaches: surgery, radio-, and chemotherapy. While the scientific and technological advancements have improved the efficacy of each of these classical approaches tremendously, and while also some new therapies have evolved including immunotherapy, the treatments apparently fail to eradicate all residual tumor cells or metastases completely. Therefore, additional means are urgently required to support or replace the conventional therapies. Hence, a variety of new approaches is currently being explored, one of which is based on the use of viruses.

Oncolytic viruses are defined by their ability to specifically kill tumor cells, but to leave the normal tissues unharmed. Their most characteristic features, thus, are their target specificity and their cytolytic capacity. Ideally, they exhibit additional features including, but not limited to, a high reproductive capacity *in vivo*, the ability to recruit uninfected neighboring cells (syncytia formation), the ability to infect both dividing and nondividing cells, a high stability *in vivo*, the inability of chromosomal integration, the lack of disease induction, and the general absence of preexisting antibodies to the virus in the host population. 

Infection of cells by viruses primarily depends on their successful entry of these cells. As a first step, virus-binding to the cell relies on the specific interaction between the viral attachment protein(s) and the cellular receptor(s). Only very few viruses have a natural preference for replication in tumor cells. Some acquired such tropism by serial passage in cell culture cells; examples include measles virus, mumps virus and Newcastle disease virus (recently reviewed in [1]), vesicular stomatitis virus [2], and reovirus [3]. Many viruses, however, lack the means for selective binding to tumor cell epitopes. To adapt these viruses for oncolytic therapy, their natural tropism needs to be altered to allow binding to tumor-specific receptors, an approach called transductional targeting. 

This review outlines the recent developments in transductional targeting of viruses. It will focus on the three strategies for retargeting of viruses that seem most promising for the development of new oncolytic viruses.  Section 2 will review the strategy through which viruses could be successfully provided new tropism by introducing targeting information into one of the viral surface proteins. As this strategy has been investigated most actively, we will limit the overview of the examples to those viruses in which the new targeting specificity could be genomically encoded. In Section 3, approaches are described by which scaffold-based modifications of viral surface proteins were applied to direct virions to new target cells, including the use of biotin or antibody-binding moieties.  Section 4 will review the use of bispecific adapter proteins as mediators of binding virions to tumor cells. Most often such adapters were simply combined with the respective viruses thereby enabling single-round infection. In some cases, these targeting devices were incorporated genetically into the virus so as to generate self-targeted agents able to independently spread through a tumor. Finally, the review will be completed with general conclusions on the current status of the field of oncolytic virotherapy and with views on its future. 

## 2. Modification of Viral Surface Proteins

**Figure 1:**
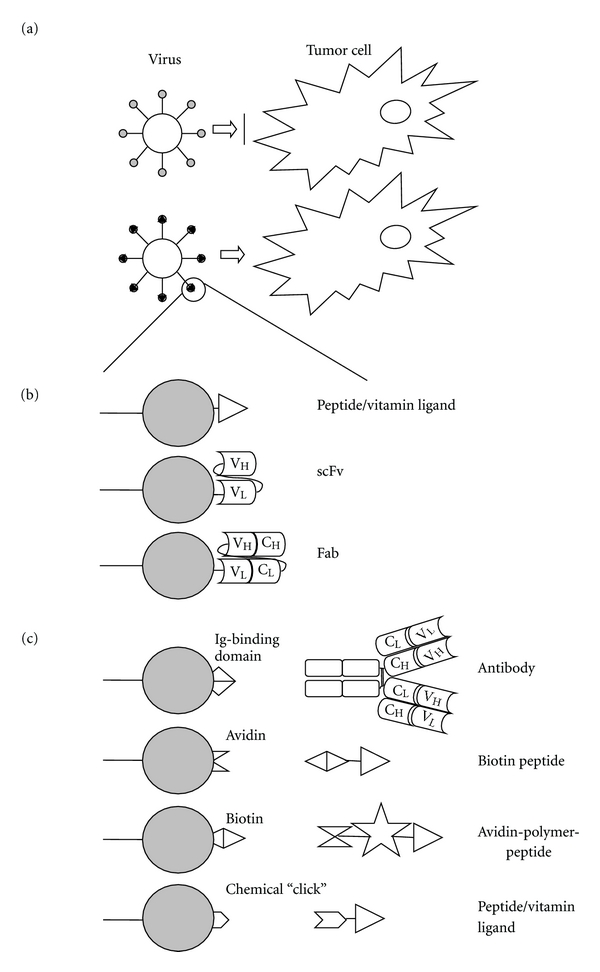
Transductional targeting through the modification of viral surface proteins. (a) Principle of redirecting viruses by the insertion of tumor-specific ligands into the viral coat, without modification of viral surface protein (upper part) no infection; (b) Schematic representation of a viral surface protein (represented by the grey-filled circle) on which tumor-binding peptides or antibodies are exposed. Ligands can be introduced at the N- or C-terminus of the protein or internally, provided that the correct folding of the viral protein and its accessibility for binding to the cell surface receptor are maintained; (c) Schematic representation of a viral surface protein on which a scaffold is exposed. The targeting ligand, examples of which are shown schematically, is then provided as a separate entity, binding on the one hand to the virion and on the other hand to the cell surface receptor of choice.

The most popular approach to generate oncolytic viruses has been by adapting their surface-exposed components. Viral surface proteins can be modified to express ligands that bind to receptors preferentially or exclusively expressed on tumor cells. Viruses can be genetically adapted to express those modifications to redirect them towards tumor cells (Figures 1(a) and 1(b)). The main advantage of this strategy is that the targeting specificity is inherent to the viral genome and will, thus, be maintained upon replication. Progeny virus is then able to infect neighboring cells harboring the target receptor, thereby establishing a multiround infection that will be maintained until no further tumor target cells remain. For this strategy, the ability to genetically modify the viral genome is crucial. Furthermore, detailed structural information about the viral surface protein to be modified is indispensible to predict at which location targeting motifs might be tolerated and will be exposed. Such motifs should not only allow binding of the modified virion to the cells but should also not be detrimental to the entry mechanism of the particular virus, not interfering for instance with the fusion of viral and cellular membrane. In addition, the targeting ligand introduced into the viral protein will have to meet size limitations. Small peptides, thus, seem the first and most obvious choice. The development of targeting strategies of viruses is, however, severely limited by a shortage of naturally existing molecules available for use as targeting ligands. Therefore, other sources of binding ligands have been investigated and incorporated into viral proteins for this purpose. These include (parts of) antibodies, like scFvs (single-chain variable fragments, composed of a fusion of the variable regions of the heavy (*V*_*H*_) and light chains (*V*_*L*_) of an immunoglobulin) or Fabs (antigen-binding fragments, composed of one constant and one variable domain from each heavy and light chain of the antibody). The feasibility of modifying viral coat proteins has been demonstrated for a number of viruses as is summarized below. 

### 2.1. Adenoviruses

Adenoviruses are among the most extensively studied viruses for oncolytic viral therapy. In a wild-type infection, adenovirus-binding to the cells is mediated by its major attachment factor, the fiber protein. Via its carboxy terminal knob domain, this protein binds to the primary cellular receptor coxsackie/adenovirus receptor (CAR). Following viral attachment, internalization is mediated through interaction of RGD motifs in the penton base with cellular *α*_*ν*_ integrins. In order to achieve CAR-independent infection by adenoviruses, the viral tropism can be modified via genetic engineering of adenovirus capsid proteins.

The list of reviews describing the development of genetically redirected adenoviruses through incorporation of ligands into viral surface proteins is numerous. In summary, heterologous peptide ligands have been successfully engineered into many adenoviral proteins, including the HI loop of the fiber, the C terminus of the fiber, the L1 loop in the hexon, and the RGD loop in the penton base and in the minor capsid protein IX. Most commonly, targeting moieties are inserted in the HI loop of the fiber knob. For this protein, the importance of the insertion site of the ligand was demonstrated when introducing a model peptide CDCRGDCFC into the knob [4]. The insertion of the ligand into three of five analyzed loops of the knob still allowed trimerization of the knob protein, and the resulting adenoviruses showed superior infectivity to that of viruses with the same peptide fused to the fiber C terminus. That the precise ligand positioning is pivotal was further demonstrated by the lack of enhancement of infectivity when the ligand-flanking linkers were extended and when tandem copies of the ligand peptide were inserted [5]. Interestingly, also antibody-based targeting could be achieved for adenoviruses by generating fiber chimeras [6] or fusions of scFvs with the capsid protein IX [7]. For the most recent reviews on transductionally targeted adenoviruses, the reader is referred to [8–11]. For adenoviruses, the possibility to combine transductional with transcriptional targeting to increase adenoviral specificity makes this group of viruses particularly interesting for future therapy; however, their strong immunogenic nature might seriously hamper their efficacy* in vivo*.

### 2.2. Paramyxoviruses-Measles Virus

The measles virus is another virus well studied for oncolytic therapy, as the attenuated measles virus strain Edmonston has the ability to selectively destroy neoplastic tissue (reviewed in [12]). Measles virus has two envelope glycoproteins: the hemagglutinin (H) attachment protein and the fusion (F) protein. Virus attachment, entry, and subsequent cell-cell fusion are mediated via the two measles receptors: CD46 and the signaling lymphocyte activation molecule (SLAM).

To improve the specificity of the infection, tumor-specific ligands have been introduced as C-terminal extensions of the H protein. A range of ligands, including both peptides and scFvs, were tolerated and, in addition, allowed the redirection of the virus to cells expressing the appropriate virus receptor. Again, the nature of the ligands was pivotal. Thus, though the length of the linkers separating the *V*_*H*_ and *V*_*L*_ domains was not of importance for scFvs to be incorporated into virions, it certainly affected the membrane fusion ability of the virus [13]. For reviews on redirected measles virus, please see [12, 14–16]. As the Edmonston strain has been used for vaccination for over 50 years now, its safety profile is impressive and might provide a good basis for future application in oncolytic therapy.

### 2.3. Herpesviruses-Herpes Simplex Virus (HSV)

Another field of active study involves the use of herpes simplex virus for tumor therapy (reviewed in [17, 18]). Herpesvirus infects cells by attachment to heparan sulfate proteoglycans, mediated by the viral glycoproteins gC and gB, followed by the interaction of the glycoprotein gD with one of two alternative protein receptors. One, designated herpesvirus entry mediator, is a member of the family of tumor necrosis factors receptors. The second involves nectin1 and nectin2, both intercellular adhesion molecules belonging to the immunoglobulin (Ig) superfamily.

Retargeting of HSV could be achieved by the insertion of ligands and scFvs into the gC and/or the gD protein, with subsequent increased infectivity of target cells expressing the appropriate virus receptor. The current strategies to redirect HSV towards tumor cells have recently been reviewed [18, 19]. For another herpesvirus, the gamma herpes virus saimiri the native binding region of the viral glycoprotein ORF51 to heparan sulphate was replaced with that of a peptide sequence interacting with somatostatin receptors, known to be overexpressed on hepatocellular carcinoma cells. The subsequent recombinant virus appeared to infect the carcinoma cells as well as the wild-type virus, while showing reduced infectivity for other cell lines. The reason for these observations is unclear [20]. In conclusion, herpesviruses remain promising as candidates for oncolytic therapy as they can be redirected to tumor cells and are considered reasonably safe due to the induction of a self-limited disease in humans. On the other hand, their wide natural tropism and the presence of viral antibodies in the human population might hamper their effectiveness *in vivo*.

### 2.4. Parvoviruses-Adenoassociated Virus (AAV)

A less frequently studied candidate for development as oncolytic agent is AAV (reviewed in [21]). AAV has a broad host cell range due to the widespread distribution of its primary cellular receptor heparan sulfate proteoglycan. The viral capsid protein is responsible for the interaction with this host cell receptor.

Transductional targeting independent of the native tropism could be demonstrated by genetically incorporating the 14-amino-acid targeting peptide L14 [22] into six different putative loops of the AAV2 capsid protein. The results showed that all mutant capsids were efficiently incorporated, that three mutants expressed L14 on the capsid surface, but that only one of these efficiently infected wild-type AAV2-resistant cell lines that expressed the integrin receptor recognized by L14. The importance of the incorporation site and of the peptide sequence was further elucidated in other studies, showing that the assembly, the generation of infectious particles, and the ability to transduce target cells depends both on the position in the capsid and on the ligand introduced [23, 24]. Successful targeting was demonstrated towards RGD [25] and towards the human luteinizing hormone receptor [24]. Due to the broad cell tropism of wild-type AAV, retargeting in combination with ablation of its natural tropism will remain crucial to develop this virus into a safe oncolytic vector.

### 2.5. Retroviruses-Murine Leukemia Virus (MuLV)

Replication-competent retroviruses have gained interest as oncolytic agents, in particular because of their high transduction efficiency (reviewed in [26, 27]). Of MuLV different classes can be distinguished, of which the host range is based on the interaction between the envelope glycoprotein and a particular cell surface receptor. While the ecotropic MuLVs are particularly capable of infecting mouse and rat cells, amphotropic MuLV infects a range of mammalian, including human cells via the widely expressed Pit-2 receptor.

Initial studies to redirect ecotropic MuLV towards human tumor cells pointed towards the importance of the interaction between the envelope glycoprotein and its original virus receptor. Despite the correct folding of chimeric envelope glycoproteins displaying scFvs, their incorporation into viral particles, and the binding of pseudotyped virus particles carrying chimeric ecotropic Env to human cells, the resulting viruses were not infectious for the targeted cells (reviewed in [28]). When expanding these studies using amphotropic MuLV, targeted infection could be achieved only when incorporating the high molecular weight melanoma-associated antigen (HMWMAA), while targeting towards the EGF [29], IGF [30], and folate [31] receptors was unsuccessful, despite the observed binding to cells expressing those receptors. It was proposed that trafficking of the virus particles to lysosomes and subsequent degradation caused the lack of infectivity, but attempts to overcome this problem by inserting a translocation domain of exotoxin A of *Pseudomonas aeruginosa* into the envelope protein, in order to translocate the virion from endosomes to the cytoplasm, were unsuccessful [29]. Clearly, the choice of receptor will be of ultimate importance for the successful targeting of retrovirus vectors towards tumor cells.

### 2.6. Poxviruses-Vaccinia Virus

Vaccinia virus has been studied for its antitumor properties already for a long time. Despite its entry into a wide range of cells, for several vaccinia virus strains, a natural preference for replication in cancer tissue has been reported. While the identity of the natural receptor is still under debate, it likely involves a widely expressed surface component, like heparan sulfate or chondroitin sulfate proteoglycans. Tumor targeting can be improved by deleting vaccinia virus genes that are necessary for replication in normal cells but not in cancer cells (recently reviewed in [32]).

To increase the specificity of their tropism, tumor-specific scFvs have been displayed on the surface of vaccinia virus particles. Targeting moieties were introduced by fusing an scFv directed against the tumor-associated antigen ErbB2 to the N-terminus of the nonessential hemagglutinin HA protein in vaccinia virus strain IHD-J [33]. Similarly, the nonessential p14 membrane-associated protein of vaccinia strain MVA could be replaced with a p14 fusion molecule carrying an inserted scFv directed against the tumor-associated antigen MUC-1 [34]. The resulting fusion proteins could be expressed, were exposed on the envelope of the recombinant virus, and were able to bind the target cells. No preferential infection of the target cells was, however, observed, likely because the recombinant viruses still contained wild-type host cell attachment proteins, providing the infection with a broad cell range. Therefore, the future challenge for the transductional targeting of vaccinia virus towards tumor cells will lie in the elimination of its natural tropism.

### 2.7. Coronaviruses-Mouse Hepatitis Virus (MHV)

The favorable characteristics of—particularly the nonhuman—coronaviruses as potential oncolytic agents have been recognized only recently. In these viruses, the spike (S) protein is responsible for receptor binding and subsequent cell entry through virus-cell membrane fusion. The aminoterminal S1 domain is required for virus-binding to the cells, and, while undergoing ordered structural changes, the S2 domain mediates fusion with the cell membrane. Infection of cells by coronaviruses depends on the expression of specific cellular receptors, which makes these viruses highly species-specific. For example, entry by MHV is mediated by the murine carcinoembryonic antigen (CEACAM1a) receptor. 

**Figure 2:**
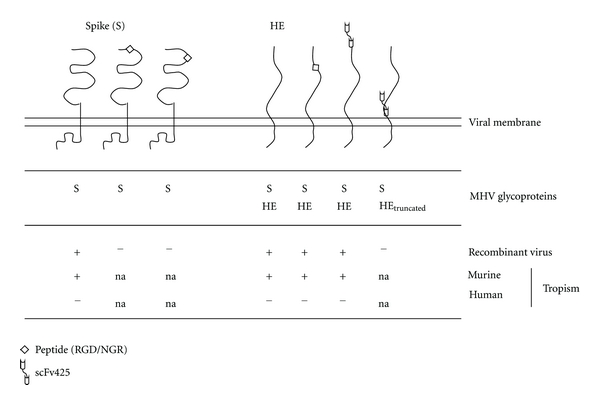
Modification of viral surface proteins for redirecting coronavirus to tumor cells. Schematic representation of MHV surface glycoproteins spike (S) and hemagglutinin-esterase (HE) and of modifications applied to redirect the virus to novel target cell antigens. Modifications tested include: insertion of small peptide ligands, including RGD and NGR, and extension with the anti-EGFR scFv425. Recombinant MHV viruses encoding such mutated S proteins or modified HE proteins (in the presence of wild type spike proteins) were generated by targeted recombination [35]. Indicated is whether the intended recombinant viruses could actually be isolated (confirmed by RT-PCR and sequencing). Also indicated is the tropism of each successfully generated recombinant virus for murine and for human cells (Verheije and Rottier, unpublished data).

Attempts to redirect coronaviruses, in particular MHV, by mutation of the viral surface proteins were unsuccessful. Incorporation of ligands, such as RGD and NGR, into various nonconserved domains in the S1 domain of the spike protein appeared to be not tolerated, as the selection of retargeted recombinant viruses based on the new binding properties of the modified spike was not successful (Figure 2; Verheije and Rottier, unpublished data). Obviously, without much knowledge of the tertiary structure of the coronavirus spike protein and of its conformational changes during cell entry, chances are high that the introduction of even small ligands affects its proper functioning. Some MHV strains carry an accessory hemagglutinin-esterase (HE) surface glycoprotein. Attempts to also use this glycoprotein for retargeting were equally unsuccessful. Though some HE gene modifications, such as insertions of small peptide ligands and terminal extensions with the anti-EGFR scFv425, could be incorporated into the viral genome, the resulting recombinant viruses were unable to redirect MHV to human tumor cells (Figure 2; Verheije and Rottier, unpublished data).

## 3. Introduction of Scaffolds into Viral Surface Proteins 

Rather than incorporating specific tumor targeting information into a viral surface protein, an alternative approach involves the incorporation of a scaffold moiety into such a protein to which subsequently various types of targeting modules can be linked (schematically depicted in Figure 1(c)). The main strategic difference relative to the previous method is that the moiety incorporated is not a tumor ligand itself but represents an attachment site for exogenously provided targeting moieties that, besides to the scaffold, also bind to the receptor of interest (compare Figures 1(b) and 1(c)). An essential operational limitation is that this targeting strategy provides viruses that can only establish single-round infection, remaining dependent on the external supply of the targeting module. Yet, it has the advantage of flexibility as the targeting device, binding always to the same, previously modified viral protein, can be changed relatively easy. Some of these strategies are based on antibody targeting, giving the opportunity to redirect the oncolytic virus to virtually every tumor surface epitope. A particular application based on this principle relies on the biotin-(strept)avidin-coupling method (reviewed in [36]).

### 3.1. Adenoviruses

For adenoviruses, single-round targeted virus particles could be generated by using the biotin-streptavidin coupling system. After incorporating a biotinacceptor peptide into the fiber, metabolically biotinylated adenovirus was coupled to an EGF-streptavidin complex and found to successfully infect EGFR expressing target cells [37]. Similarly, a biotin-polyethylene glycol (PEG)-EGF conjugate coupled to an avidin-modified adenovirus could redirect the virus to a nonnative receptor [38]. In another study, small protein ligands capable of selective binding to human IgG and IgA were incorporated as model ligands for tropism-modified adenoviruses. Viable viruses that had genetically incorporated scaffolds into their fiber gene could be rescued and were, after incubation with antibodies, able to enter cells displaying the Fc receptor on their surface [39]. Recently, it has been demonstrated that specific chemoselective modification of the adenoviral particle could also function as a scaffold for targeting devices. By metabolic incorporation of noncanonical monosaccharides and amino acids in adenoviral particles, conjugation with a folate targeting motif in combination with a taxoid was achieved. Initial results demonstrated increased toxicity in vitro [40].

### 3.2. Adenoassociated Virus

The Ig-binding fragment of protein A was tested as a possible scaffold to redirect AAV. The fragment was successfully introduced into the capsid protein providing a versatile platform for antibody-mediated AAV targeting [41]. In a more recent study, a biotinacceptor peptide was incorporated into AAV particles. Subsequent biotin labeling of the viruses with the biotin ligase BirA and attachment of an RGD peptide to target integrins resulted in a significant increase in the transduction of endothelial cells, demonstrating again the feasibility of this approach [42].

### 3.3. Togaviruses-Sindbis Virus

Sindbis virus has inherent oncolytic properties and has been studied quite extensively as an oncolytic virus (reviewed in [43]). One of the surface proteins on mammalian cells to mediate the Sindbis virus infection is the laminin receptor, which is overexpressed on various human tumors. The envelope protein E2 of Sindbis virus is responsible for receptor binding.

To increase the specificity of the infection, researchers combined the introduction of ligands into the viral envelope with the use of targeting molecules. To this end, virus particles were generated which contained the IgG-binding domain of protein A inserted into their envelope protein E2. When combined with antibodies that bound to specific surface antigens on nonsusceptible cells, the chimeric virus was able to infect these otherwise refractory cells [44]. A comparable combination approach was taken by introducing Ig-binding domains as N-terminal extensions of the E2 glycoprotein. After adding species-matched antibodies, Fc receptor-positive cell lines could be successfully infected [45]. 

### 3.4. Murine Leukemia Virus

Also for MLV, studies were performed to introduce the IgG-binding domain of protein A to enable modular use of antibodies of various specificities for vector targeting. By inserting this binding domain into the hinge region of the viral envelope protein virions were generated that were capable of capturing anti-HER2 antibodies. Subsequent efficient binding of the virus-antibody complex to HER2-positive target cells and enhancement of transduction of these cells was observed [46].

## 4. Transductional Targeting of Viruses Using Bispecific Adapters

### 4.1. Bispecific Adapters

**Figure 3:**
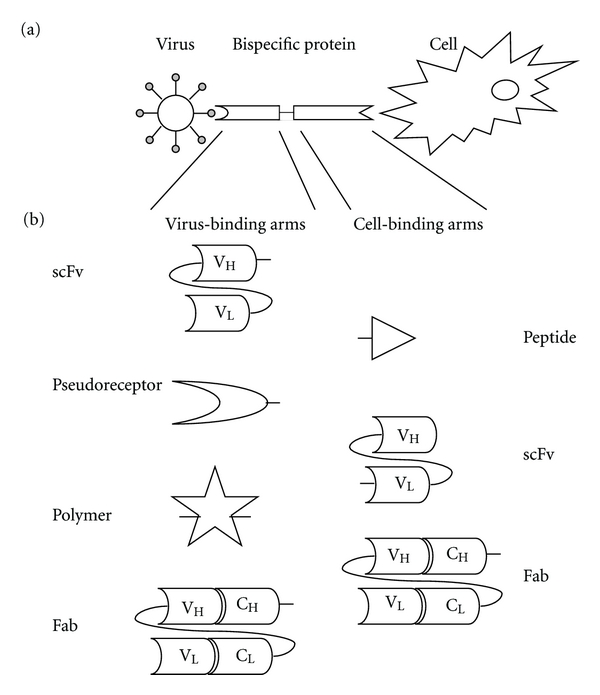
Bispecific adapter targeting principle and composition. (a) Principle of targeting viruses towards tumor cells using bispecific adapters. (b) Typical examples of virus-binding moieties (left) and cell-binding moieties (right) of bispecific adapters. In theory, all combinations of virus-binding and cell-binding arms are possible. An overview of the composition of the bispecific adapters used to target particular viruses to tumor cells is provided in Table 1.

An elegant strategy currently employed to target viruses towards tumor cells makes use of bispecific adapters. Such proteins consist of two domains (“arms”), one binding to the virion, the other to a cell surface epitope of interest, thereby enabling indirect interaction of virus and tumor cell. The composition of the adapter proteins can vary greatly, depending on the design of the arms. The virus-binding domains that have been used include soluble receptor fragments (so-called pseudoreceptors), polymers like PEG, (parts of) antibodies, including scFvs or Fabs. Moieties that have been applied for cell-binding are natural peptide or vitamin ligands for receptors, and again scFvs or Fabs directed against a cell epitope of interest. The specificity for two different antigens is achieved either by joining the arms together chemically or by combining the two moieties in one fusion protein, often with a flexible linker, the targeting arm typically being at the C-terminus. The principle of redirecting viruses towards nonnative cells using bispecific proteins is shown in Figure 3(a), with typical examples of the two domains being depicted in Figure 3(b). In Table 1, an overview is provided of combinations of arms in bispecific adapter proteins actually generated to target viruses to tumor cells.

### 4.2. Advantages and Disadvantages of Bispecific Adapters for Targeting

The use of adapters to redirect viruses towards tumor cells has several advantages over the introduction of targeting or scaffold moieties into viral attachment proteins. First, no detailed structural information is required about the viral surface proteins, as the manipulation of these proteins is not required. Second, as the size of the targeting part of the adapter protein seems less crucial than when introducing this moiety into a viral protein, the choice of targets can easily be expanded by using (parts of) antibodies. In this way, the selection of targeting receptors becomes virtually unlimited, as antibodies can be generated relatively easy, once the receptor of interest has been identified. Third, as adapter proteins are straightforward to construct, expanding the repertoire of target receptors becomes relatively easy. Finally, the binding of adapter proteins to the virion has, at least in some cases, been reported to ablate the virus' natural tropism, which is especially useful when the oncolytic virus of choice has a preference for normal cells in the host.

There are also disadvantages of using bispecific proteins in targeting oncolytic viruses. As bispecific proteins are artificial polypeptides composed of parts that do not occur linked together naturally, their proper biogenesis with independent folding of both moieties and efficient secretion may be impaired. Moreover, unless expressed by the oncolytic virus itself, production and purification of the adaptors is a challenge.

### 4.3. Application of Bispecific Adapters

To redirect oncolytic viruses towards tumor cells, bispecific proteins can be applied in two ways. First, after recombinant production or chemical synthesis, the proteins can be precomplexed with virions before applying them *in vitro* or *in vivo*. A major drawback of this approach is, however, that it allows single-round infection only; progeny virus will not be able to infect neighboring cells as the amount of adapter protein will be limiting. Consequently, the use of such adapter-precomplexed viruses *in vivo* is likely to be restricted to local, rather than systemic application. It will probably also require repeated administration of high doses of the adapter proteins. Little is known about the potential risks of such approach. 

As an alternative, the genetic information for the adapter protein can be incorporated into the viral genome. When properly expressed, this ensures the local production of the targeting device together with the progeny virus in the infected cell. This approach will enable multiround infection and lateral spread of the oncolytic virus. The time span and, hence, the efficacy of this kind of therapy will be limited by the emergence of immunity against the bispecific protein and/or the virus. The feasibility of this strategy depends on the availability of a genetic modification system to introduce the adapter-encoding gene into the viral genome as an additional expression cassette. While such modification systems are currently available for most viruses, the capacity of the genome to accept such insertions can be limited; hence, the size of the targeting moiety might be restricted. Finally, the genetic stability of such recombinant viruses might be an issue, in particular when the adapter protein is used to ablate the natural tropism of the virus.

The feasibility of using adapter proteins for oncolytic viral therapy has been explored for a number of viruses. Below we first present an overview of the studies in which targeting to tumor cells was performed by coadministration of viruses and adaptor proteins (Section 4.3.1). Thereafter, we discuss the studies describing genetic targeting of oncolytic viruses generated by the incorporation of genes coding for bispecific proteins into the viral genome (Section 4.3.2).

#### 4.3.1. Single-Round Transductional Targeting Using Bispecific Adapters

**Adenoviruses**

To redirect adenoviruses to tumor cells, neutralizing antiknob fiber antibodies have been used extensively. The first demonstration of their potential application for retargeting of these viruses to a nonadenovirus cellular receptor was in 1996, when such antibodies were chemically conjugated to folate and shown to mediate infection of folate-receptor expressing cells [47]. Many nonnative receptors have since been targeted by conjugating an antiknob antibody fragment to either ligand peptides or antibody domains directed against a cellular receptor. This resulted in successful targeting towards the EGF receptor (EGFR) [48, 49], FGF receptor [50–52], integrins [53, 54], EGP-2 (also known as EpCAM) [55, 56], the melanoma-associated antigen HMWMAA receptor [57], carbonic anhydrase IX protein G250 [58], CD40 [59, 60], various organ- and tumor homing peptide receptors [61], mesothelin MSLN [62], prostate-specific membrane antigen PSMA [54, 63], VEGFR2 [54], Ly-6D [64], and Tie2 receptors [54].

Similar approaches have been explored using antibodies directed against other adenoviral proteins. Thus, Fabs directed against the penton base of the fiber in combination with targeting ligands, such as EGF, IGF, and TNF*α* could mediate the infection of target cells expressing the appropriate receptors [65]. Also Fabs directed against the hexon protein chemically linked to Fabs specifically binding to an antigen highly overexpressed on human hepatocellular carcinoma were successfully applied to redirect adenoviruses to a nonnative receptor [66].

Another strategy successfully employed for the same purpose made use of pseudoreceptors. In this approach, bispecific proteins were generated by fusion of a soluble form of CAR (sCAR) to EGF [67, 68], to the Fc region of human IgG1 [69], and to scFvs against ErbB2 [70] and CEA [71].

In yet another approach, polymers were exploited as targeting ligands in a single round fashion. Here, adenovirus particles were coated to inhibit their natural tropism after which ligands, including peptides and scFvs, were attached. Several types of polymers have been used [72–77], including polyethylene and metacrylamide derivatives, to successfully target adenoviruses to FGF2 [78], RGD [79], TNF*α* [80], and the HER2 receptor [81]. It has been proposed that adenoviral coating with polymers might have enhanced potential for systemic delivery, as it prolongs the viral plasma half-live and reduces the hepatotoxicity *in vivo* [80].

Finally, another type of bispecific molecule based on the binding ability of the Gla domain of coagulation factor X to the hexon was exploited for targeting. Upon fusion of Gla to scFv proteins, increased infection of tumor cells by adenovirus could be observed. However, the anticipated reduction in liver transduction was not observed [82].

**Adenoassociated Viruses**

AAV has a broad host cell range due to the widespread distribution of its primary cellular receptor heparan sulfate proteoglycan. To achieve a more specific infection, a bispecific Fab was tested of which one arm recognized the cell-surface integrins *α*IIb*β*3 while the other bound to the AAV capsid [83]. Targeting this way did not inhibit downstream steps required for productive infection. Moreover, a decrease of infection of normally permissive cells was observed, indicating that the bispecific protein was able to ablate the normal tropism.

**Herpesviruses-Herpes Simplex Virus**

HSV binding to the cell is mediated by several widely expressed cell surface receptors, including nectin1. HSV was successfully redirected to the EGFR by means of a soluble adapter protein comprising the N-terminal domain of nectin1 fused to an scFv directed against EGFR [84]. Adapter-mediated entry was, however, promoted by the presence of heparan sulfate proteoglycans on cells, which are also required for wild-type HSV infection.

**Paramyxoviruses-Newcastle Disease Virus (NDV)**

In the avian paramyxovirus NDV, the hemagglutinin-neuraminidase (HN) protein is responsible for sialic acid receptor attachment, while the F protein mediates the fusion of viral envelope and cellular membrane. NDV has oncolytic properties by nature; however, it has a broad cell tropism due to the widespread occurrence of sialic acids on many cells. To narrow its specificity, the use of bispecific adapter proteins has been investigated. Preincubation of NDV with a recombinant bispecific protein composed of an scFv against HN that blocks the native receptor binding site and the interleukin-2 peptide clearly enhanced the specificity of the virus [85] and reduced its side-effects when applied systemically *in vivo* [86].

**Coronaviruses-Mouse Hepatitis Coronavirus (MHV) and Feline Infectious Peritonitis Virus (FIPV)**

The first demonstration of retargeting of coronaviruses was achieved by exchanging the viral spike ectodomains. Thus, felinized MHV (fMHV) [87] and murinized FIPV (mFIPV) [35] were generated in which the murine viruses carried the feline S ectodomain and *vice versa*. These otherwise highly species-specific recombinant viruses were able to cross species barriers; fMHV had acquired feline cell tropism but completely lost its murine cell tropism while the opposite was true for mFIPV. To extend the species tropism of nonhuman coronaviruses towards human tumor cells, bispecific adapter proteins were generated. Proteins composed of a bispecific scFv directed against both the feline spike protein and the EGFR could mediate FIPV and fMHV infection of EGFR-expressing human cancer cells, with subsequent syncytia formation typical of a productive coronavirus infection [88].

Subsequent studies to redirect murine coronavirus MHV to human tumor cells were based on an adapter protein that consisted of a pseudoreceptor, composed of the N-terminal domain of murine CEACAM1a (soluble receptor; soR), fused to an scFv directed against the EGFR [89] or to the EGF ligand [90]. Again, such adapter proteins could mediate EGFR-specific entry of MHV into human cancer cells. However, in contrast to many of the previous examples, no ablation of the natural tropism of the virus was observed.

#### 4.3.2. Multiple-Round Transductional Targeting Using Bispecific Adapters

To overcome the major drawback inherent to single-round targeting, a number of investigations focused on the expression of the bispecific adapters from the viral genome in order to allow the recombinant viruses to produce their own targeting device and sustain the infection. The feasibility of this approach has so far only been demonstrated for some adenoviruses and coronaviruses.

**Adenoviruses**

To redirect adenoviruses to nonnative surface receptors, conditionally replicating adenoviruses (CRAds) seem to be the viruses of choice, due to their selective replication in tumor cells. The first experiments demonstrating the ability to redirect CRAds towards tumor cells were performed using dual-virus mixtures consisting of a CRAd and an adenovirus secreting a bispecific adapter protein consisting of a fusion between the soluble CAR receptor and the EGF ligand [91]. Dual virus infections resulted in increased oncolytic activity *in vitro* and improved therapeutic efficacy *in vivo*.

Subsequently, CRAds were engineered to express the bispecific adapter proteins by themselves. Van Beusechem et al. [92] developed such a CRAd encoding a bispecific protein composed of the anti-EGFR scFv 425 and antifiber knob scFv s11. The resulting virus AdΔ24-425S11 produced the bispecific protein 425-s11 during replication in cancer cells, yielding progeny virus with enhanced infectivity and oncolytic properties on EGFR-positive, CAR-deficient tumor cells. However, in addition to infection mediated by EGFR, the virus retained its capacity to infect cells through binding to the native receptors CAR and integrins. To abolish the native tropism, mutations were introduced that eliminated CAR and integrin binding [93], resulting in a recombinant virus with a strictly EGFR-dependent targeting profile and reduced replication in EGFR-negative cells. Both viruses displayed similar oncolytic potency in cell lines and tissue specimens [93]. Also when applied in a mouse model by intrajugular or intramuscular injection, the native tropism of adenoviruses appeared to be reduced after the removal of both the CAR and integrin-binding sites [94]. Strikingly, however, when expressing the soluble CAR-EGF targeting moiety from a CRAd rather than from a dual virus system, its oncolytic potential was severely impaired [95], suggesting that the expression of biologically active proteins can be counterproductive to virus replication.

To overcome the biosynthetic differences between the bispecific proteins translated and secreted via the ER-Golgi route and the adenovirus with translation in the cytoplasm but assembly in the nucleus, an elegant strategy was developed by tagging of the adenovirus fiber and the scFv each with a synthetic leucine zipper-like dimerization domain [96]. Tagging of the proteins with the zipper peptide sequences preserved both the trimerization capability of the adenovirus fiber and the recognition of the EGFR by the zipper-scFv protein, but, most importantly, it gave rise to receptor-specific infection of the target cells.

Several studies have shown the feasibility of using bispecific proteins for redirecting adenoviruses towards target cells *ex vivo* or *in vivo* in laboratory animal models, including [71, 92, 97–102]. Although quite effective, these studies were all based on a two-component strategy, requiring the mixing of virions with bispecific proteins before administration. To our knowledge, no *in vivo* studies have yet been performed using recombinant adenoviruses expressing a bispecific adapter from their viral genome to establish whether they have superior targeting and cell-killing abilities.

**Coronaviruses**

To generate self-targeted coronaviruses, initially the coding sequence for a bispecific adapter protein composed of the soluble mCEACAM1a receptor linked to a His-tag was incorporated into the MHV viral genome by targeted recombination, creating the virus-designated MHVsoR-His [103]. The presence of this additional expression cassette was tolerated and the resulting recombinant viruses indeed expressed the adapter protein. Inoculation of target cells expressing the artificial His-receptor on their surface showed the recombinant viruses to be able to establish a multiround, receptor-dependent infection. Furthermore, extensive cell-cell fusion and rapid cell killing of infected target cells was observed, demonstrating the possibility of generating genetically redirected coronaviruses [103].

**Figure 4:**
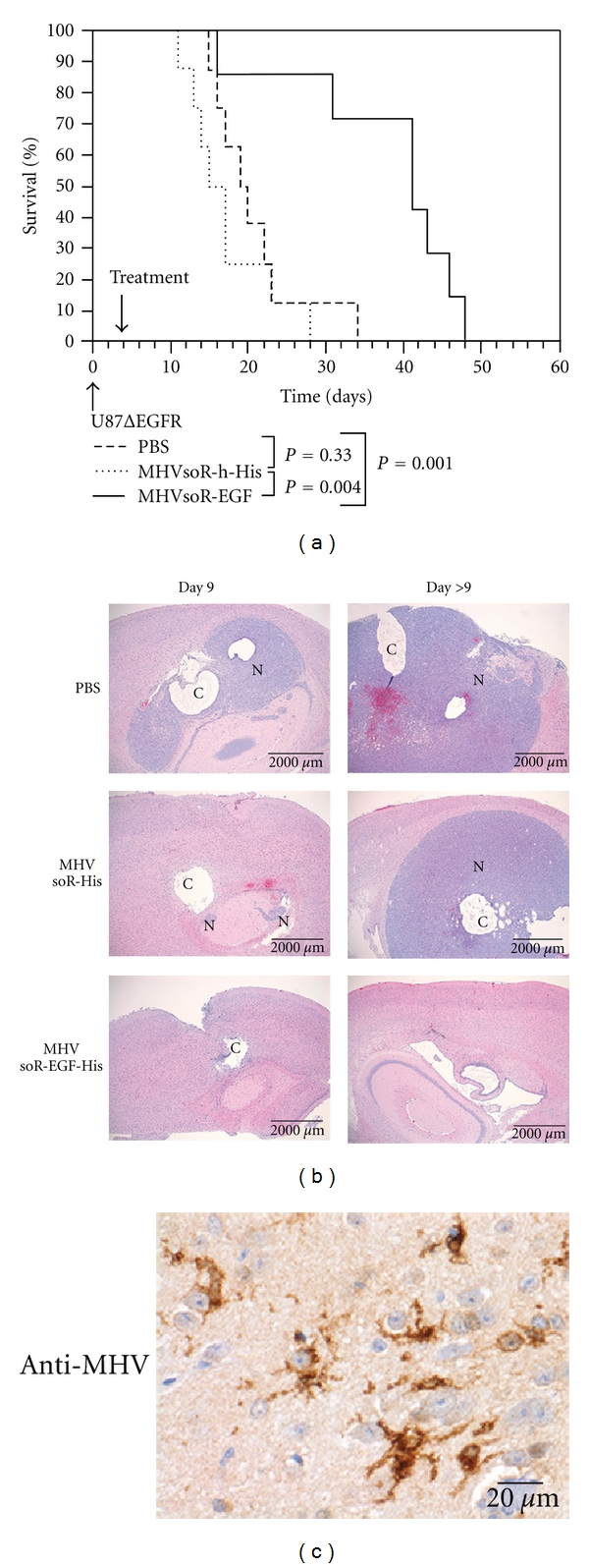
*In vivo* oncolytic activity of murine coronavirus MHV. Mice with established intracranial U87ΔEGFR tumors were treated with MHV genetically redirected to the EGFR (MHVsoR-EGF-His), the His-receptor (MHVsoR-his), or with PBS. (a) Survival curves. (b) Histopathological analysis of brains at day 9 posttreatment and at the day of euthanasia (day > 9). Large neoplasms and cystic structures are indicated by “N” and “C,” respectively. (c) Immunostaining of brains after treatment with MHVsoR-EGF-His using polyclonal anti-MHV antibodies (Copyright © American Society for Microbiology, Journal of Virology, Vol. 83, No. 15, P. 7507-16, 2009, DOI 10.1128/JVI.00495-09).

**Figure 5:**
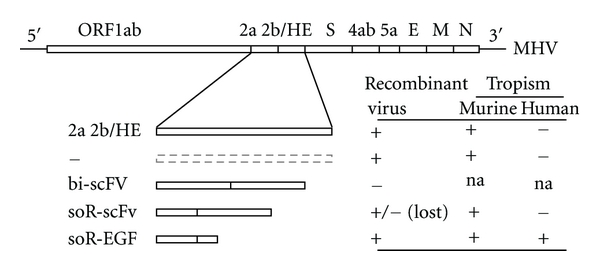
Introduction of bispecific adapter cassettes into the coronaviral genome. Schematic representation of the MHV genome and of the bispecific expression cassettes introduced herein. The plus-strand RNA genome contains, from 5′ to 3′, the polymerase precursor gene (ORF1ab), the accessory genes 2a and 2b/HE, the S gene, the nonessential genes 4ab and 5a, and the genes encoding the virion proteins E, M, and N. In the recombinant viruses, the gene cluster 2a + 2b/HE was replaced with an expression cassette downstream of the translation regulation sequence for protein 2a. Recombinant MHV viruses were generated by targeted recombination [35]. Indicated is whether the particular recombinant virus could be isolated, as confirmed by RT-PCR on virus RNA. In addition, the ability of such viruses to infect murine and human cells is depicted (Verheije and Rottier, unpublished data). Abbreviations of adapters as specified in the text: “na”: not applicable.

The expression cassette was subsequently extended by inserting the sequence encoding the EGF peptide between that of the soluble receptor and the His-tag [90]. Again, the generated recombinant MHVsoR-EGF-His thereby acquired the ability to cause multiround infection of otherwise nonsusceptible, EGFR-expressing cell cultures *in vitro*, with subsequent efficient cytolytic activity [90]. More importantly, the redirected virus demonstrated oncolytic capacity also *in vivo* in an orthotopic U87dEGFR xenograft mouse model. Survival rates of the mice were significantly longer when the tumor-bearing animals were treated with MHVsoR-EGF-His than after treatment with control virus MHVsoR-His or with PBS (Figure 4(a)). In none of the MHVsoR-EGF-His treated mice-recurrent tumor load could be detected, demonstrating the strong oncolytic capacity of such viruses *in vivo* [90] (Figure 4(b)). Despite the impressive oncolytic effect *in vivo* of the redirected MHV, replication of MHV in non-tumor tissue of the natural host was observed (Figure 4(c)), presumably because the natural tropism of MHV was not ablated.Further experiments demonstrated that the composition of the bispecific protein is of critical importance for the success of generating recombinant oncolytic coronaviruses. In particular, viable recombinant coronaviruses expressing a bispecific scFv from an additional expression cassette in the viral genome could not be rescued (Figure 5; Verheije and Rottier, unpublished data). Subsequent introduction of a bispecific gene encoding the soR fused to a scFv against the EGFR did generate viable viruses; however, such viruses were genetically highly unstable, loosing the foreign gene usually already within one passage (Figure 5; Verheije and Rottier, unpublished data). As successful incorporation of other, even larger, foreign genes at the same position in the MHV genome has been reported (including for example the gene-encoding luciferase [104]), the instability of the scFvs is likely due to their particular sequence composition rather than to their size.

In conclusion, oncolytic coronaviruses expressing a soluble receptor that is C-terminally extended with a peptide ligand have great potential for oncolytic therapy. By expanding the targeting repertoire through exchange of peptide ligands, coronaviruses can probably be redirected towards various tumor epitopes, provided that the binding and fusion ability of the viral proteins are maintained. As murine coronaviruses display great species specificity in their infection, ablation of the natural tropism will probably not be required, making MHV a safe candidate oncolytic agent for use in other mammals, including humans.

## 5. Conclusions and Perspectives

Appealing as the idea of oncolytic virotherapy may be, its realization is still disconcertingly remote. What this overview emphatically reveals is that the field of retargeting of viruses for therapeutic use is in its early infancy. In fact, for the majority of the viruses studied, scientists are still struggling with the most fundamental aspects of changing their cell tropism. Robust platforms for retargeting have actually not been established yet for any of the viruses. On the positive side, the feasibility of retargeting was, at least *in vitro,* demonstrated for an increasing number of viruses in the past years. Clearly the most attractive goal will be to generate oncolytic viruses that carry the retargeting information in their genome. Only then will the viruses be able to sustain their replication in the tumor tissue, irrespective of the retargeting principle used, that is, whether through the modification of the viral attachment protein or through expression of an adapter protein. However, (pre)clinical data on the efficacy—let alone safety—of such transductionally targeted viruses *in vivo* are very limited. With one exception (a transductionally and transcriptionally targeted adenovirus [105]), none of such genetically modified, tropism modified oncolytic viruses have entered phase I clinical trial. This makes it virtually impossible to compare the viruses reviewed here. Thus, many hurdles have yet to be overcome before new oncolytic viruses will reach the clinic. Below, some of the important future tasks and challenges for the field are summarized.

One major challenge at the base of the idea of oncolytic virotherapy is the availability of suitable target receptors on tumor cells. Ideally, such receptors are unique or highly overexpressed in order to provide sufficient specificity for the infection. Recent developments in the proteomics field have already recognized various proteins that are overexpressed in tumor cells as compared to normal tissue and many more will hopefully be identified. It remains, however, questionable whether truly unique tumor surface proteins exist. This stresses the need to increase specificity of oncolytic viruses in other ways. This can be achieved, for example, by combining transductional targeting with either transcriptional targeting or attenuation of the viral genome, both increasing tumor-specific replication. In transcriptional targeting, viral genes essential for replication are placed under control of a tumor-specific promoter—which is particularly feasible for DNA viruses—or under the control of an IRES element in the case of RNA viruses. Attenuation of the viral genome might be achieved by the deletion of viral genes that eliminate functions dispensable in tumor cells, but not in normal tissue. The feasibility of combining both transductional and translational targeting has already been demonstrated for DNA viruses, including adenovirus, while for RNA viruses investigations rather focus on the transductional targeting of attenuated viruses. 

The natural tropism of the therapeutic virus is another aspect which needs to be taken into account with regard to safety. Ablation of the native tropism might be required for those viruses naturally infecting humans, to prevent the infection of normal tissue, but also when the virus has a preference for binding, for instance, to blood substances or when it exhibits hepatic tropism, both being a major cause of loss of infectious virus *in vivo*. The use of nonhuman viruses for oncolytic therapy gains interest, as such viruses are usually nonpathogenic for humans and, in addition, no preexisting antibodies circulate which might limit their efficacy. However, when adapting to the new host, these viruses might also pose a risk, as was reviewed in [106]. 

In order to achieve effective eradication of all tumor cells, a desirable characteristic of oncolytic viruses is their ability to cause sustained, multiround infection. This can be achieved by viruses genetically redirected through the incorporation of tumor-binding ligands and those having incorporated a bispecific adapter into their viral genome The stability of such recombinant viruses might, however, be a matter of concern, in particular when the targeting protein is required to ablate the natural human tropism. In general, DNA viruses are considered to be more stable than RNA viruses in which, in addition, the mutation rate is relatively high. 

Irrespective of the origin of the virus, immunity induced upon (repeated) viral treatment against viral antigens but also against foreign proteins like the bispecific adapters expressed from the viral genome might limit the effectiveness of the therapy. To shield viruses from antibodies, polymers might be used to coat the virion [107]. Yet, this might compromise the binding of the virus to tumor cells and can technically only be performed upon application and not during lateral spread of the virus. Other ways to increase the delivery of oncolytic virus to tumor cells, especially when applied systemically, might be the use of carrier cells, which have the ability to home to the tumor [108].

In conclusion, transductionally targeted viruses may provide a much needed tumor-specific therapy, but researchers will have to face, besides the technological challenges, a delicate balance between safety and effectiveness during development of such new viruses for clinical use. Yet, despite all problems and concerns, the importance of the ultimate goal of winning the fight against cancer warrants the sacrifice of all the energy and creativity needed for its realization. 

## Figures and Tables

**Table 1:** Overview of the composition of bispecific adapters used to reroute viruses for oncolytic purposes.

Adaptor protein moiety binding to		Targeting demonstrated for
Virion	Cell	
Antibody (scFv or Fab)	Antibody (scFv or Fab)	Adenovirus
		Adenoassociated virus
		Coronavirus
Antibody (scFv or Fab)	Ligand peptide	Adenovirus
		Paramyxovirus

Soluble/pseudo receptor	Antibody (scFv or Fab)	Adenovirus
		Herpesvirus
		Coronavirus
Soluble/pseudo receptor	Ligand peptide	Adenovirus
		Coronavirus

Polymer	Antibody (scFv or Fab)	Adenovirus
Polymer	Ligand peptide	Adenovirus
